# Directed Evolution of Phi Class Glutathione Transferases Involved in Multiple-Herbicide Resistance of Grass Weeds and Crops

**DOI:** 10.3390/ijms23137469

**Published:** 2022-07-05

**Authors:** Elisavet Ioannou, Anastassios C. Papageorgiou, Nikolaos E. Labrou

**Affiliations:** 1 Laboratory of Enzyme Technology, Department of Biotechnology, School of Applied Biology and Biotechnology, Agricultural University of Athens, 75 Iera Odos Street, 11855 Athens, Greece; elis_ioan@hotmail.com; 2 Turku Bioscience Centre, University of Turku and Åbo Akademi University, 20520 Turku, Finland; anapap@utu.fi

**Keywords:** glutathione transferase, DNA shuffling, structural analysis, catalysis, thermal stability, inhibition potency

## Abstract

The extensive application of herbicides in crop cultivation has indisputably led to the emergence of weed populations characterized by multiple herbicide resistance (MHR). This phenomenon is associated with the enhanced metabolism and detoxifying ability of endogenous enzymes, such as phi class glutathione transferases (GSTFs). In the present work, a library of mutant GSTFs was created by in vitro directed evolution via DNA shuffling. Selected *gstf* genes from the weeds *Alopecurus myosuroides* and *Lolium rigidum*, and the cereal crops *Triticum durum* and *Hordeum vulgare* were recombined to forge a library of novel chimeric GSTFs. The library was activity screened and the best-performing enzyme variants were purified and characterized. The work allowed the identification of enzyme variants that exhibit an eight-fold improvement in their catalytic efficiency, higher thermal stability (8.3 °C) and three-times higher inhibition sensitivity towards the herbicide butachlor. The crystal structures of the best-performing enzyme variants were determined by X-ray crystallography. Structural analysis allowed the identification of specific structural elements that are responsible for k_cat_ regulation, thermal stability and inhibition potency. These improved novel enzymes hold the potential for utilization in biocatalysis and green biotechnology applications. The results of the present work contribute significantly to our knowledge of the structure and function of phi class plant GSTs and shed light on their involvement in the mechanisms of MHR.

## 1. Introduction

The phenomenon of multiple herbicide resistance (MHR) refers to tolerance manifestation in most chemical compounds currently utilized in post-emerging weed control that also exhibit limited similarities in their structure and function [1,2]. Weed growth is the most important bio-factor causing yield reductions in the global agricultural industry. Herbicides are the most effective answer to the problem, though their intensive application has led to the emergence of numerous resistant weed populations, thus threatening the sustainable intensification of crop cultivation [3,4,5,6,7]. There are currently over 500 cases of herbicide resistance [8].

MHR is an undeniable problem in noxious weeds, such as black-grass (*Alopecurus myosuroides*) and annual ryegrass (*Lolium rigidum*), that compete with cereal crops, particularly in Europe and Australia [9,10]. In these weeds, MHR is associated with enhanced metabolism and detoxifying properties of their endogenous enzymes, including P450 monoxygenases (CYP450s) and phi class glutathione transferases (GSTFs), which abide by the non-target site resistance mechanism (NTSR) [11,12]. NTSR includes mechanisms that obstruct lethal herbicide doses from reaching their specific target site [13].

GSTs are a multifunctional superfamily of enzymes, distributed in all major kingdoms of living organisms. They are long established to catalyze the conjugation of the tripeptide glutathione with diverse electrophilic and hydrophobic compounds, resulting in their modification to display higher solubility and less toxicity [14]. The phi and tau classes of GSTs, which are primarily found in plants, exhibit substrate specificity and are responsible for herbicide detoxification. The phi class of GSTs (GSTFs) has been documented to exhibit high activity towards chloroacetanilide and thiocarbamate herbicides [15]. This class involves a variety of genes that can be induced due to environmental factors or biotic stresses. Hence, the expressed GSTFs exhibit peroxidase activity and play a role in the biosynthesis and transport of secondary metabolites [16]. GSTF induction also occurrs as a result of treatments that invoke plant defense reactions, osmotic stress and exposure to extreme temperatures [17,18,19]. GSTFs expression can be enhanced by herbicide safeners as reported in cereals treated with compounds that enhance herbicide tolerance [20,21].

The implication of GSTs in herbicide resistance was initially reported in the 1970s for the herbicide atrazine [22]. Thereafter, publications on the GST-mediated resistance to multiple herbicides based on an increase in GST activity and/or gene expression were reported [11,23,24,25,26,27,28]. Notably, detoxifying enzyme levels are greater in domesticated cereal crops than in their competing weeds; thus, these enzymes are largely responsible for differences in herbicide metabolism and selectivity [29,30,31,32,33]. Overexpression of GSTFs has been identified in *A. myosuroides* and *L. rigidum* weeds that have demonstrated herbicide tolerance. However, the lack of increased detoxifying enzymatic ability in these enzymes due to their kinetic profile has led to the conclusion that they possess a direct regulatory role in cell metabolism by controlling the accumulation of protective flavonoids. The key role of GSTFs in MHR was further highlighted when their inhibition by GST inhibitors helped in restoring herbicide control in *A. myosuroides* [11,30,31,32,33]. Furthermore, herbicide resistance of *A. myosuroides* and *Lolium spp.* towards the herbicide flufenacet is correlated with enhanced GST activity [34,35]. This herbicide has been widely utilized in the control of emerging multiple-resistant weeds in Europe [35].

GSTs that belong to the phi class are dimeric enzymes, comprised of two identical subunits. Each subunit consists of two domains, a smaller thioredoxin-like N-terminal domain (residues 1–78) and a larger C-terminal domain (residues 92–213) that is formed only by α-helixes. Residues of the N-terminal domain residues assist the formation of a GSH binding site (G-site), whereas hydrophobic residues of the C-terminal domain form the hydrophobic substrate binding site (H-site) [14]. All the studied GSTFs belong to Ser-GSTs, since the ancestral cysteine in their active site has been replaced by serine at position 12, thus exhibiting the motif of Ser-Thr-Asn in the G-site of the helix α1 [16,30,33]. Ser-GSTs usually exhibit peroxidase activity in addition to glutathione conjugation reactions, which may assist in herbicide detoxification [36].

Protein engineering is a powerful tool that aims to generate novel proteins/enzymes with industrial, therapeutic and research potential. It enables the development of molecular tools for the manipulation of detoxifying enzymatic properties and it can also provide insights into the evolution of resistance mechanisms in the xenobiotic metabolism [37,38,39,40,41,42]. GSTs are designated as a versatile tool for protein engineering due to their catalytic multifunction, substrate specificity, structural characteristics, ease in heterologous expression and stability [38,42]. There are multiple examples of directed evolution of plant GSTs that provide compelling results regarding their structural profile and catalytic properties. Dixon et al. [15], using DNA shuffling of tau class GST genes from *Zea mays* along with further site-directed mutagenesis, managed to produce mutants that exhibited up to 29-fold enhanced detoxifying ability for the herbicide fluorodifen. Furthermore, DNA shuffling of homologous tau class GST genes from *Glycine max* resulted in mutants with unusual allosteric kinetics and enhanced detoxifying potential towards this herbicide [40]. In another work, a library of tau class GSTs from abiotic stress-treated *Phaseolus vulgaris* and *Glycine max* plants was constructed, thus producing a novel enzyme with increased glutathione hydroperoxidase activity and unusual kinetics towards 1-chloro-2,4-dinitrochlorobenzene (CDNB) [41]. More recently, DNA shuffling of three homologous tau class glutathione transferases resulted in a GST variant with enhanced catalytic activity towards the herbicide alachlor [42]. This enzyme variant was explored for the development of an optical biosensor for alachlor determination.

In the present work, an in vitro directed evolution approach was implemented via DNA shuffling for homologous recombination of selected *gstf* genes [30,31] from the cereal crops *Triticum durum* and *Hordeum vulgare*, as well as the weeds *Alopecurus myosuroides* and *Lolium rigidum*. The work aimed at the creation of a library of detoxifying enzymes with improved catalytic properties and structural stability that could be further utilized in green biotechnology applications.

## 2. Results and Discussion

### 2.1. Shuffling of Parental GSTF Genes Encoded in A. myosuroides, L. rigidum, T. durum and H. vulgare and Activity Screening

Alignment of the parental GSTFs from *A. myosuroides, L. rigidum, T. durum* and *H. vulgare* [30,31] showed 88% and 72% sequence homology at the protein and DNA level, respectively. Despite the high homology in primary structures, their kinetics and catalytic properties differ significantly, allowing an interesting research perspective. For example, the catalytic constants k_cat_ of *Hv*GSTF and *Td*GSTF are significantly higher than those of *Lr*GSTF and *Am*GSTF (Table 1). There was a 5- and 7.5-fold difference in the k_cat_ values and the catalytic efficiencies (k_cat_/K_m_) between the less active *Lr*GSTF and the more active *Ta*GSTF, respectively. Therefore, the selected group of phi class GSTs represents an ideal model for studying structure/function relationships through directed evolution approaches.

**Figure 1:**
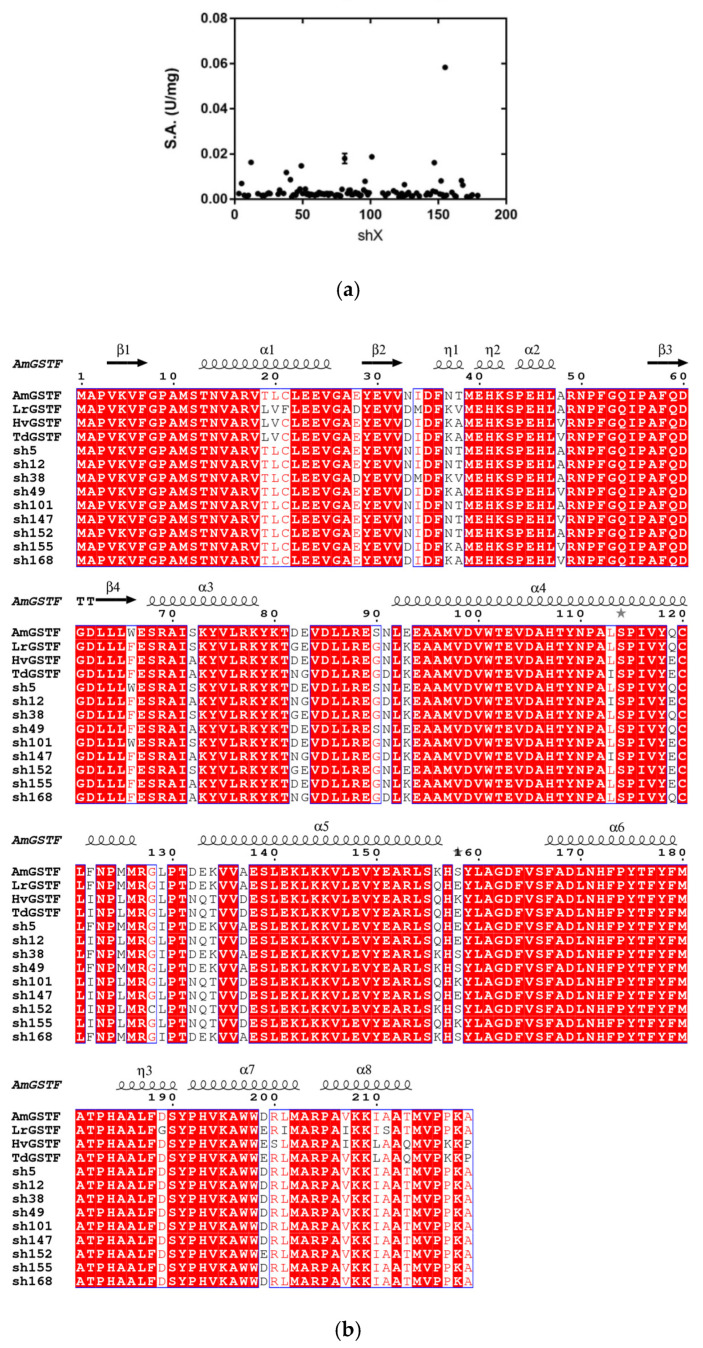
(**a**) Activity screening of colonies obtained by DNA shuffling. Only the colonies that displayed detectable activity with the substrate system CDNB/GSH are depicted. (**b**) Protein sequence alignment of parent GSTFs (NCBI accession numbers *Am*GSTF: CAA09192.1, *Lr*GSTF: CCO25537.1, *Hv*GSTF: AAL73394.1, *Td*GSTF: VAH13982.1) and selected variants obtained by DNA shuffling. Shaded areas displayed a similarity score value over 0.7 (0 to 1 range) based on their physicochemical properties. The letters are colored based on similarity using the “Thermal” option in ESPript 3.0 [43]. The secondary structure of *Am*GSTF (PDB code: 6RIV) is shown at the top. Alpha helices and beta strands are represented as helices and arrows, respectively. Beta turns are marked with TT. Initial alignment was accomplished by Clustal Omega [44].

DNA recombination of four *gstf* genes produced a library of novel chimeric enzymes. After in vitro recombination of gene fragments, a single PCR amplicon was produced and cloned into the pETite™ C-His vector. Activity screening was achieved for 180 randomly picked colonies using the substrate system CDNB/GSH (Figure 1a). 

Approximately 60% of the assayed colonies exhibited glutathione transferase activity, indicating that the DNA shuffling protocol resulted in a satisfying percentage of catalytically active enzymes. Seven colonies (sh12, sh49, sh101, sh147, sh152, sh155 and sh168) that exhibited high activity were initially selected. The recombinant plasmids were purified and DNA sequenced. The results showed the creation of chimeric *gstf* genes consisting of parental fragmented regions, thus highlighting their successful recombination (Figure 1b and Figure S1). According to the gene sequence, the shuffled enzymes appear to have been evolved from the *Am*GSTF through reassembly of different fragments derived from the other three enzymes. Therefore, the *Am*GSTF can be considered as the parent enzyme.

The parent GSTFs along with the seven selected reassembled clones were expressed in either BL21 (DE3) pLysS or Rosetta™ 2 (DE3) pLysS *E. coli* cells and were purified by Ni-IDA-Sepharose affinity chromatography with yields ranging from 90 to 99% and purity > 95% as evaluated by SDS-PAGE (Figures S2 and S3).

### 2.2. Kinetic Studies

The kinetic parameters of the wild type along with the seven shuffled GSTFs were measured using the substrate system CDNB/GSH in 0.1 M phosphate buffer, pH 6.5, equilibrated at 37 °C. The analysis showed that the wild-type GSTFs exhibited comparable kinetic behavior and parameters to that reported by Georgakis et al., 2020, where kinetic analysis was performed at 25 °C [30]. We selected the use of 37 °C instead of 25 °C for enhancing the sensitivity of the assay in mutants with low catalytic activity. Steady-state kinetic analysis with GSH as a variable substrate and CDNB at a fixed concentration complied with the Michaelis–Menten model; however, the kinetics using CDNB as a variable substrate exhibited allosteric behavior with positive cooperativity (Table 1, Figures S4 and S5). A well-known property of some members of phi and tau class GSTs is their allosteric kinetics towards the xenobiotic substrates [30,39,41]. It has been reported that allosteric kinetics is the consequence of intersubunit structural communication of the dimeric GSTs, where the binding of one CDNB molecule in one H-site promotes, through the dimer interface, the transmission of conformational changes in the structure of the H-site of the neighbor subunit [39,41].

The positive cooperativity of the enzymes, observed in the present study, indicates their higher sensitivity to changes in substrate concentration, despite the poorer binding response at low substrate concentrations [45]. Such a kinetic profile may offer an advantage in the mechanisms of cell detoxification. However, there are also members of phi and tau class GSTs that have been previously reported to obey Michaelis–Menten kinetics [16,46,47,48,49,50].

The results listed in Table 1 indicate a relative low variation in the K_m_ or S_0.5_ parameters of the shuffled GSTFs, in agreement with the wild-type enzymes; however, their catalytic constants k_cat_ displayed considerably larger variations. GSTFs from the crops *T. durum* and *H. vulgare* displayed significantly higher turnover numbers and k_cat_/K_m_ or k_cat_/S_0.5_ ratios compared to *Lr*GSTF and *Am*GSTF (Table 1). The shuffled enzymes sh49 and sh168 exhibited k_cat_ and k_cat_/K_m_ values similar to the wild-type enzymes *Lr*GSTF and *Am*GSTF, while the rest displayed substantially higher catalytic constants (Table 1). Among the selected enzymes, sh101 and sh155 exhibited the highest improvement in k_cat_ values (185.9 ± 1.8 min^−1^ and 197.7 ± 3.2 min^−1^, respectively), corresponding to an approximately 5-fold improvement, compared to the wild-type *Am*GSTF enzyme (35.9 ± 1.7 min^−1^). Furthermore, the sh101 enzyme exhibited lower S_0.5_ and K_m_ values towards CDNB, leading to significant improvement (7–8 times) in catalytic efficiency compared to the parent enzyme *Am*GSTF. It is noteworthy that the sh101 enzyme displayed the highest catalytic efficiency towards CDNB (k_cat_/S_0.5_) among all four parent enzymes.

According to the gene sequence, the shuffled enzymes sh101 and sh155 have been evolved from the parent enzyme *Am*GSTF and were created by reassembling of *Am*GSTF and *Hv*GSTF/*Td*GSTF fragments. The variants sh101 and sh155 possess 11 and 17 mutations respectively, compared to the wild-type *Am*GSTF. Sh101 possesses mutations derived from fragments of either *Hv*GSTF or *Td*GSTF that were combined primarily at its C-terminal region. Sh155 carries the same C-terminal mutations, as well as some other mutations in the region 34 to 38 near its N-terminal region (Figure 1b).

**Figure 2:**
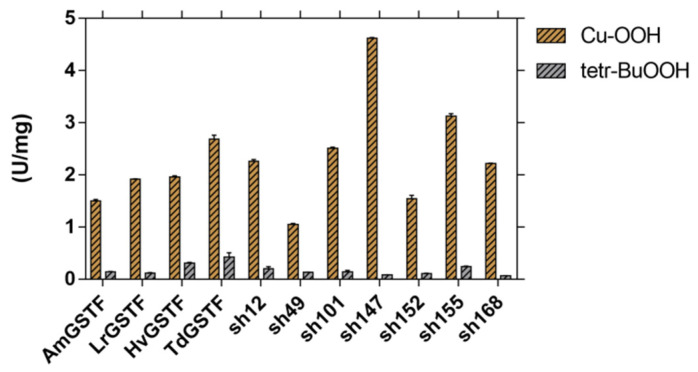
The glutathione-dependent peroxidase activity of the parent GSTFs and shuffled enzyme variants. The glutathione-dependent peroxidase activity was assayed using as substrates cumene hydroperoxide (CuOOH) and tert-butyl hydroperoxide (tert-BOOH).

The purified enzymes were also assessed for glutathione-dependent peroxidase activity (Figure 2). It is well established that members of phi class GSTs [16,23,30,47] exhibit significant glutathione peroxidase activity towards cumene hydroperoxide. The parent GSTFs correspond to Ser-type GSTs, since their active site ancestral cysteine has been replaced by a Ser residue at position 12; thus, they display the STN motif (Ser12-Thr13-Asn14) in α1 helix of the G-site [16,30]. Ser-type GSTs exhibit high hydroperoxidase activity that contributes to their detoxifying role [36] and has been associated with the MHR phenomenon by assisting in an antioxidant protective mechanism against toxic organic hydroperoxides, which are formed as a result of abiotic stress caused by herbicides [24].

### 2.3. Determination of Butachlor’s Half-Maximal Inhibitory Concentration (IC_50_) for the GSTF Library

Previous investigations in our lab [30,31] have shown that the parent enzymes display restricted ligandin function and are able to bind a narrow range of pesticides with high affinity. For instance, chloroacetanilide herbicides appear to bind with high affinity among different families of pesticides [31].

**Figure 3:**
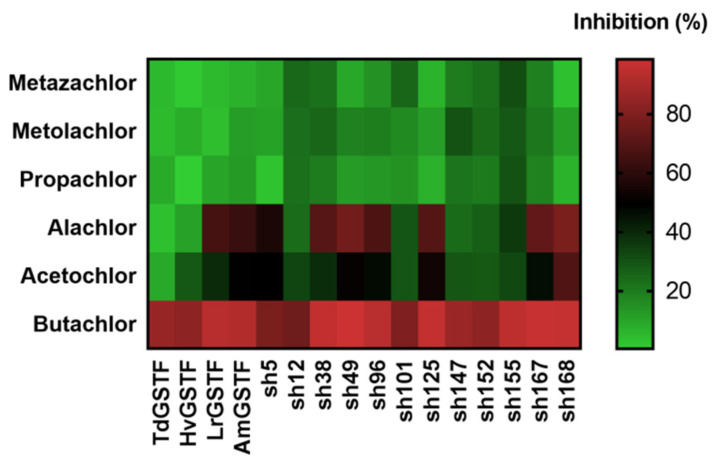
Heat map depiction of the inhibition (%) of parent GSTFs (*Am*GSTF, *Lr*GSTF, *Hv*GSTF, *Td*GSTF) and shuffled enzyme variants (sh12, sh49, sh101, sh147, sh152, sh155, sh168) by chloroacetanilide herbicides (50 μM).

**Figure 4:**
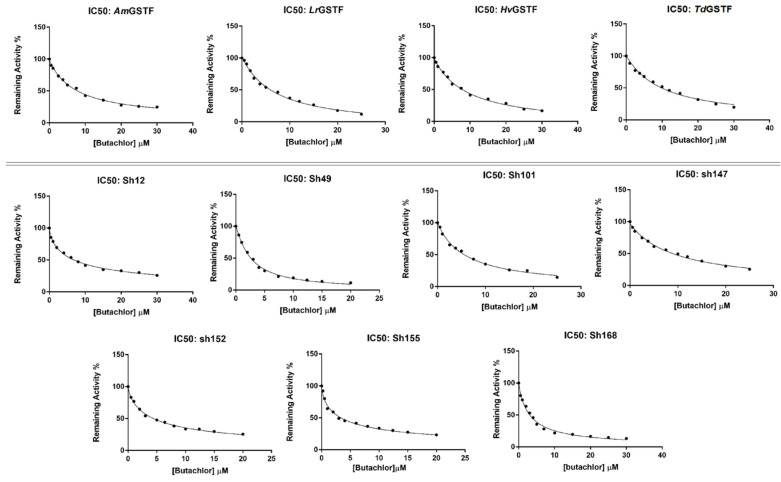
Dose-response inhibition curves of the parent GSTFs (*Am*GSTF, *Lr*GSTF, *Hv*GSTF, *Td*GSTF) and shuffled enzyme variants (sh12, sh49, sh101, sh147, sh152, sh155, sh168) by the herbicide butachlor for the determination of IC_50_ value. The measurements were performed in triplicate and the data represent the mean ± SD (N = 3).

In order to assess the effect of recombination on the ligandin function of the enzyme variants, six different chloroacetanilide herbicides were tested as inhibitors (Figure 3). The results showed that between these herbicides, butachlor displayed substantial potency towards all the tested enzymes. Dose-response measurements allowed the determination of IC_50_ of butachlor towards all the enzymes [51]. The values estimated among the wild-type enzymes did not show significant differences in inhibition potency among weed (*Lr*GSTF and *Am*GSTF) and crop (*Td*GSTF and *Hv*GSTF) enzymes. However, it was estimated that sh49, sh155 and sh168 exhibited up to 3-fold lower IC_50_ values than the crop GSTFs (Table 2, Figure 4). This provides a significant prospect for utilization in several biotechnological applications and developments. For example, the enzyme variant sh155 could potentially be a promising candidate for the development of a butachlor biosensor, since it combines low IC_50_ values with high catalytic activity.

### 2.4. Evaluation of GSTF Thermal Stability

**Figure 5:**
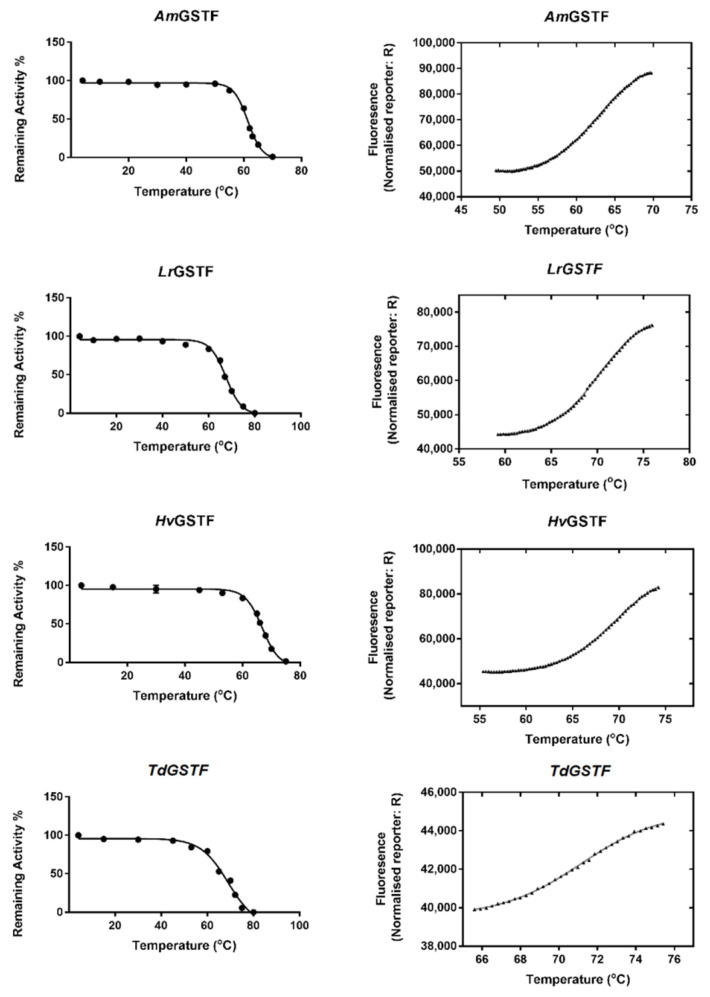
Thermal stability studies of the parent enzymes (*Am*GSTF, *Lr*GSTF, *Hv*GSTF, *Td*GSTF). Left column: thermal inactivation curves. The remaining enzyme activities (%) were measured after heat treatment of each enzyme at the indicated temperatures (°C) for 5 min. Right column: Differential scanning fluorimetry normalised curves for the determination of the melting temperature (T_m_). The measurements were performed in triplicate and the data represent the mean ± SD (N = 3).

**Figure 6:**
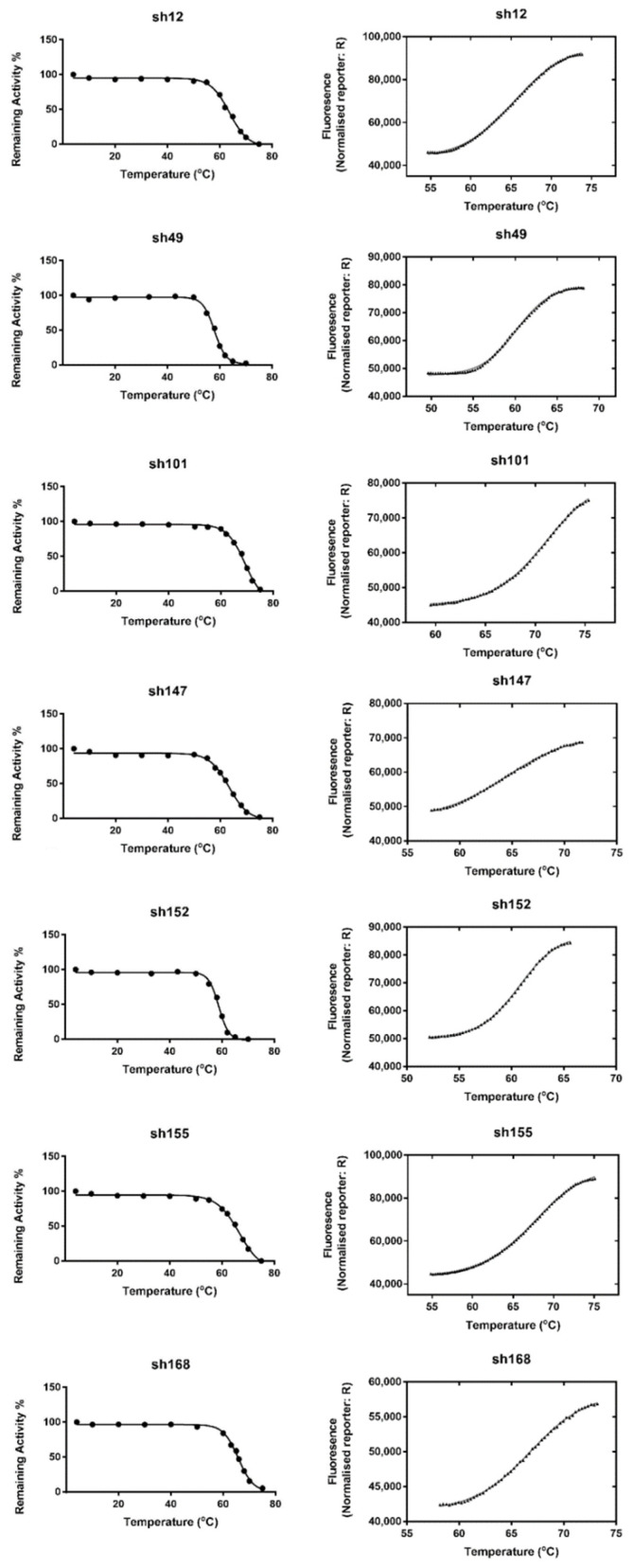
Thermal stability studies of the DNA shuffled enzyme variants (sh12, sh49, sh101, sh147, sh152, sh155, sh168). Left column: thermal inactivation curves. The remaining enzyme activities (%) were measured after heat treatment of each enzyme at the indicated temperatures (°C) for 5 min. Right column: Differential scanning fluorimetry normalised curves for determination of the melting temperature (T_m_). The measurements were performed in triplicate and the data represent the mean ± SD (N = 3).

The thermal stability of both parent and shuffled enzyme variants was assessed to evaluate the effect of mutations and recombination on structural integrity. The thermal stability was measured by employing two complementary methods, namely differential scanning fluorimetry and thermal inactivation studies (Figure 5 and Figure 6). Melting temperatures (T_m_ values) were estimated by differential scanning fluorimetry in four assay replicates and the results are listed in Table 3. Interestingly, all the enzymes showed T_m_ values that exceeded 60 °C, indicating high thermal stability. The wild-type enzymes *Lr*GSTF, *Td*GSTF and *Hv*GSTF exhibited T_m_ values of approximately 70 °C (Figure 5); however, the T_m_ of *Am*GSTF was found to be significantly lower (62.8 ± 0.04 °C). Among all enzymes, the sh101 and sh155 enzyme variants exhibited the highest T_m_ values (Table 3, Figure 6), suggesting that the mutations introduced to these have been beneficial not only for affording improved kinetic properties but also for providing structural stability. In particular, the T_m_ values of sh101 and sh155 enzyme variants were significantly increased by 8.3 and 5.2 °C compared to the parent enzyme *Am*GSTF.

For the measurement of the half maximal thermal inactivation temperature (T_50_), the enzymes were incubated for 5 min at different temperatures (4–80 °C) and their remaining catalytic activity was determined by enzyme assays. The results showed that the T_50_ values ranged between 58 °C and 69 °C (Table 3). In general, the measured T_50_ values are in good agreement with those estimated by differential scanning fluorimetry, although slight differences (1–3 °C) were observed (Table 3).

The high thermal stability of the enzymes measured in the present study are aligned well with previously published work on tau class plant GSTs [39,41,52,53,54], supporting the idea that the plant specific tau and phi class GSTs display high thermostability compared to the well-known mammalian counterparts, underlining their suitability for developing biotechnological applications.

### 2.5. Overall Description of the Crystal Structure of sh101 and sh155 Enzyme Variants

To further understand the results of the kinetics and stability analysis and in order to put the data in a structural context, the crystal structures of the two most interesting enzyme variants, sh101 and sh155, were determined by X-ray crystallography. Structural analysis was employed to identify structural elements important for k_cat_ regulation, thermostability and inhibition by chloroacetanilide herbicides.

The structures of sh101 and sh155 enzyme variants were resolved at 1.87 and 2.00 Å resolution and compared to the wild-type *Am*GSTF enzyme structure that has been recently reported [30]. The sh155 enzyme shares 92.24% sequence identity with *Am*GSTF and 97.26% with the sh101 enzyme variant. Amino acid sequence alignments of the sh101 with the parent enzyme *Am*GSTF showed 94.98% identity (Figure S1).

**Figure 7:**
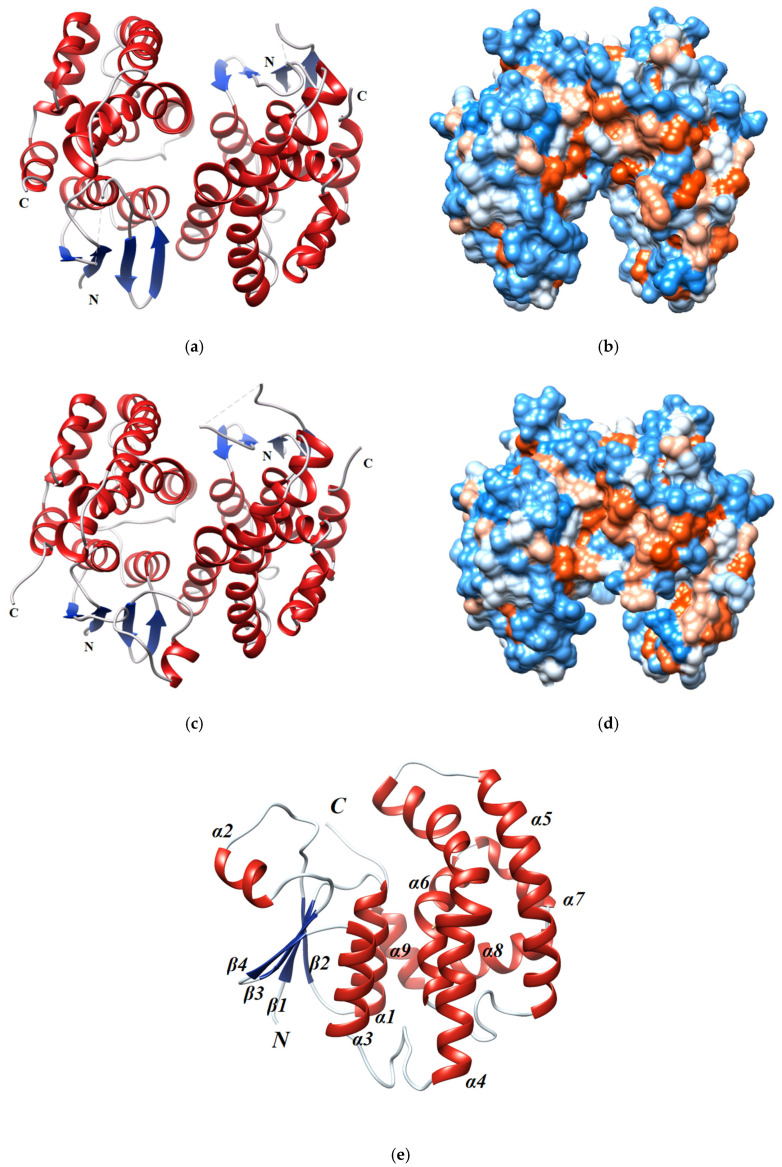
Crystal structure of the sh101 and sh155 enzyme variants. (**a**,**c**) Ribbon representation of the dimer protein (sh101 and sh155, respectively). The α-helixes (red) and β-strands (blue) are depicted. (**b**,**d**) Surface hydrophobicity of the homodimer protein. Hydrophilic areas are shaded in a blue color range and hydrophobic areas in an orange-red color range. The figures were created by UCSF Chimera [55]. (**e**) Ribbon representation of the monomer of sh155 variant. The α-helixes (red) and β-strands (blue) are labelled.

Sh101 and sh155 were crystallized with two and three molecules in the asymmetric unit, respectively. Each molecule of the sh101 and sh155 enzyme variants adopts the common GST-fold and consists of 216 residues, when the first methionine of its protein sequence is removed. Each subunit is composed of a smaller thioredoxin-like N-terminal domain (residues 1–78) and a larger C-terminal domain (residues 92–213) that is formed only by α-helixes (Figure 7).

The N-terminal domain retains an α/β structure similar to that reported for other plant GSTs [14,30,36,38,40,41,42,56,57,58]. This domain consists of a four-stranded β-sheet formed by β1 (Val4 to Phe7), β2 (Tyr29 to Val32), antiparallel β3 (Ala57 to Asp60), β4 (Leu63 to Leu65) sheets located between three larger α-helixes (α1: Thr13 to Glu24, α2: Pro44 to Arg49 and α3: Glu67 to Lys78). The sequence between the β2 and β3 strands where the α2 helix is located exhibits significant distortion due to high flexibility [14,40]. This part has not been modelled in sh101 because of the high flexibility and lack of electron density. In sh155, high flexibility was observed in the same region in two of the molecules, while in the third one sufficient density was found that enabled the building of the entire loop between β2 and β3. The C-terminal domain consists of six α-helixes (α4: Leu92-Arg127, α5: Gln133-Gln156, α6: Phe167-Ala181, α7: Pro183-Ser190, α8: Pro192-Ala203, α9: Pro205-Thr213). Helixes α4 and α5 are positioned almost parallel to each other, while α6 and α8 are connected by the smaller α7 helix similar to previously studied GSTF structures [16,30,50,56] (Figure 7e).

**Figure 8:**
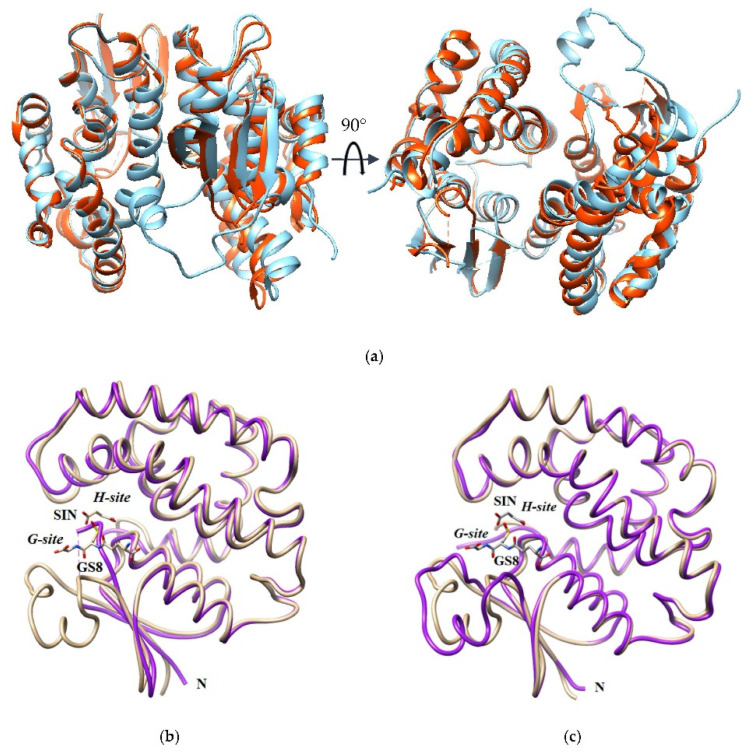
(**a**) Superposition of the sh101 (dark orange) and sh155 (blue) dimeric structures. The letter N represents the N-terminal site. (**b**) Superposition of the monomers of sh101 (purple) and *Am*GSTF (PDBid 6riv) (beige). The succinic acid (SIN) and glutathione sulfenic acid (GS8) molecules bound to the *Am*GSTF structure are shown as sticks and colored according to the atom type. (**c**) Superposition of the monomers of sh155 (purple) and *Am*GSTF (beige). The succinic acid (SIN) and glutathione sulfenic acid (GS8) molecules bound to the *Am*GSTF structure are shown as sticks and colored according to the atom type.

Superposition of sh101 and sh155 with *Am*GSTF (PDB id 6riv) showed 0.458 Å and 0.478 Å RMSD (Root Mean Square Deviation), respectively, indicating subtle structural differences between them (Figure 8). Notably, the structure of the α2 helix (chain A: Ile35-Pro50, chain B: Phe37-Asn49) in the sh101 enzyme displayed significant changes compared to the wild-type *Am*GSTF. The α2 helix contains Phe36, an important residue that contributes to the formation of the H-site. Previous investigations have established the role of Phe35 (Phe36) in the modulation of k_cat_ by affecting product release in the GSTF1-1 enzyme from maize [59].

#### 2.5.1. Structural Elements That Contribute to k_cat_ Regulation

Homology modelling was performed in order to complement the structural data of the available crystallographic structures of sh101, sh155, *Am*GSTF and *Lr*GSTF [30,31]. The crystal structure of the parental enzyme *Am*GSTF in complex with the ligands glutathione sulfenic acid (GS8) and succinic acid (SIN) was used as a template for the construction of sh12, sh49, sh147, sh152 and sh168 models. The sequence identity is approximately 91% to the template structure (Table S3), suggesting reliable homology modelling.

**Figure 9:**
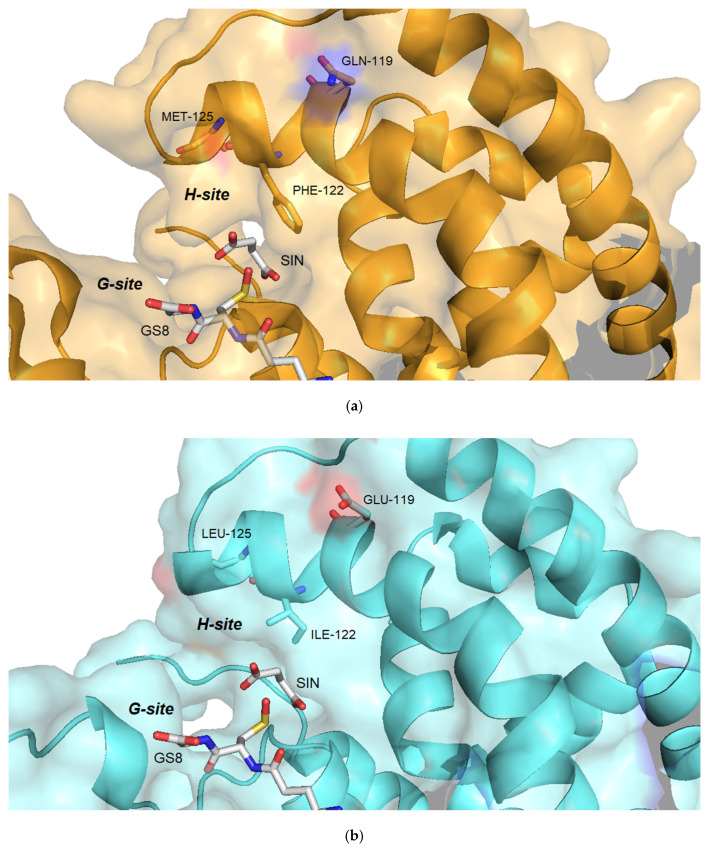
Amino acid residues at positions 119, 122 and 125 in the H-site of *Am*GSTF (**a**) and sh155 (**b**). The mutations in the sh155 are: Gln119Glu, Phe122Ile and Met125Leu. The succinic acid (SIN) and glutathione sulfenic acid (GS8) molecules bound to the *Am*GSTF structure are shown as sticks and colored according to the atom type. The figures were created with PyMol [61].

All the amino acid residues that contribute to the formation of the G-site (Ser12, Lys42, Gly53, Gln54, Pro56, Glu67, Ser68, Arg69) in the sh101 and sh155 variants, as well as in the other enzymes, are totally conserved. Consequently, the K_m_ values for GSH fall within a narrow range and are similar to those of the parent enzymes (Table 1). On the other hand, large diversity was observed in the regions that contribute to the H-site formation. For instance, the region Tyr118-Arg127 has three substitutions in sh101 sequence (Gln119Glu, Phe122Ile, Met125Leu), as a result of the replacement of the C-terminal part of the α4 helix by the sequence derived from the crop-type GSTFs (*Td*GSTF and *Hv*GSTF). These substitutions in the H-site are probably related to the differences in their kinetic characteristics, despite their high overall homology. Phe122 is of particular importance as it has been reported to be involved in van der Waals interactions with the xenobiotic substrate in the H-site [30] (Figure 9). This residue has been substituted in the sh12, sh101, sh147, sh152 and sh155 structures by the non-polar and less bulky residue Ile, found in the crop-type parent enzymes *Td*GSTF and *Hv*GSTF. Similarly, Met125, which is present in the structure of *Am*GSTF, *Lr*GSTF, sh49 and sh168, has been substituted by a non-polar Leu125 in the sequences of *Td*GSTF, *Hv*GSTF, sh12, sh101, sh147, sh152 and sh155. The role of the residue at position 125 appears to be less important, since its orientation lies towards the solvent and lacks any significant interaction with the substrate. Kinetic analysis of the enzymes containing Ile122 (*Td*GSTF, *Hv*GSTF, sh12, sh101, sh147, sh152, sh155) showed that they display higher k_cat_ values. It is noteworthy that the H-site region Tyr118-Arg127, in the variants sh49 and sh168, has not been replaced by the respective crop-type sequence (*Td*GSTF and *Hv*GSTF) and consequently the k_cat_ values are very similar to those of the weed-type GSTs (*Am*GSTF, *Lr*GSTF), confirming the crucial role of amino acid at position 122 in k_cat_ regulation.

The structure of the α2 helix (chain A: Ile34-Pro51, chain B: Phe36-Asn50) in sh101 displayed significant flexibility compared to the wild-type *Am*GSTF. The α2 helix contains important residues (Lys42, Gly53, Gln54) that contribute to the formation of the G-site. Previous investigations have established the crucial role of the α2 helix in k_cat_ modulation in the GSTF1-1 enzyme from maize [59] and in human GSTP1-1 enzyme, by affecting product release [60]. Furthermore, the α2 helix is involved in the induced-fit mechanism that accompanies substrate binding and catalysis. The structure of the α2 helix in the sh101 and sh155 variants appears to be more flexible and adopts different conformation compared to the *Am*GSTF parent enzyme. These conformational variations and the enhanced flexibility, in the sh101 and sh155 variants, appear to be restricted in the *Am*GSTF parent enzyme because of the new interactions formed by Lys42, Gly53, Gln54 side chains and GSH.

**Figure 10:**
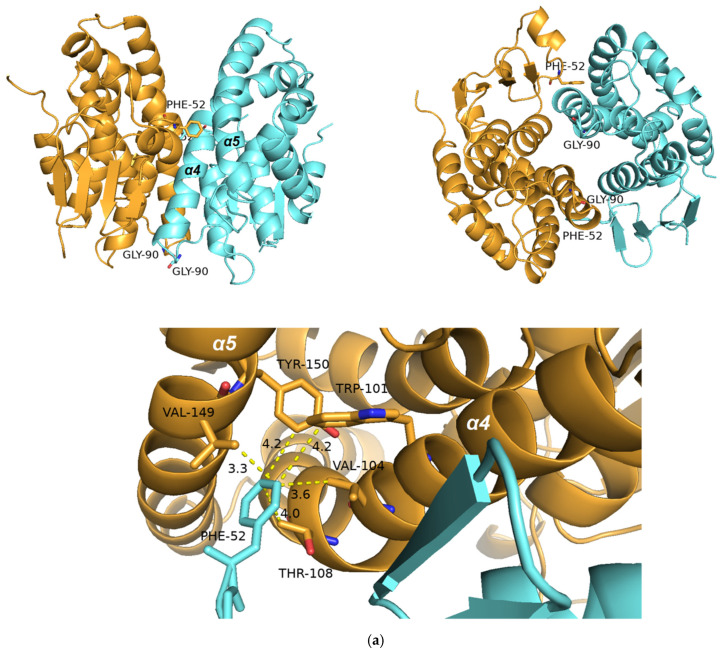

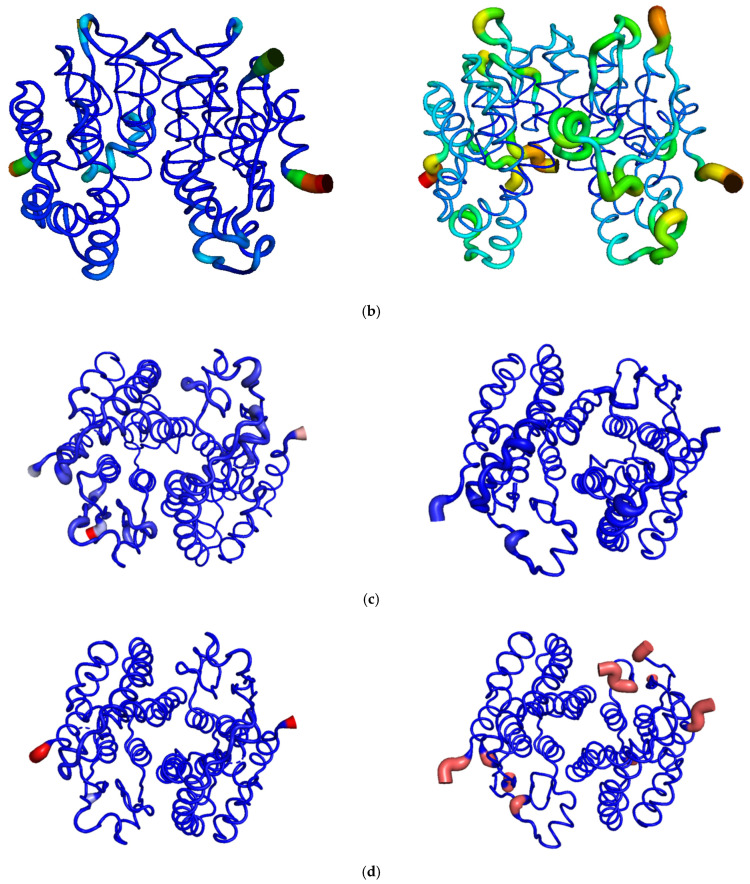
(**a**) Intersubunit structural communication between α2 helix and the kinked large α4 helix in the sh155 enzyme. The amino acid residue at position 90 and the lock-and-key forming residues (Phe52, Trp101 and Val104) are shown and labelled. Amino acid side chains are shown as sticks. (**b**) Structural flexibility along the polypeptide chain in the parent *Am*GSTF (left) and the sh155 enzyme variant (right). Regions of low mobility have a thinner backbone radius, whereas regions of higher mobility have a thicker backbone radius. The plots were produced by PyMol [61]. (**c**) Plot of deformation energy along the polypeptide chain of *Am*GSTF (left) and sh155 (right). (**d**) Plot of atomic fluctuation along the polypeptide chain of *Am*GSTF (left) and sh155 (right). The (**c**,**d**) plots were produced by DynaMut web server [62]. The deformation/fluctuation magnitude is represented by thin to thick tubes colored blue (low) to red (high).

As already discussed, the allosteric kinetics in GSTs is the consequence of intersubunit structural communication of the dimeric structure, where the binding of one CDNB molecule in one H-site transmits, through the dimer interface, conformational changes to the H-site of the neighbor subunit [39,41,52,53,59]. Interestingly, the enzyme variants with Hill coefficient n_H_ > 1.28 (sh49, sh168), including the parent enzyme *Am*GSTF (n_H_ = 1.5), appear to exhibit significantly lower k_cat_ values compared to sh101 and sh155, suggesting that the positive cooperativity negatively affects the catalysis (Table 1). Analysis of the structures reported here, and taking into account the results from previous investigations [39,41,52,53,59], supports the conclusion that the observed allosteric kinetics is the consequence of intersubunit structural communication between α2 helix and the large kinked α4 helix (Leu92-Met126, Figure 1) that crosses the entire structure (Figure 10). In all highly active variants, including sh101 and sh155, the mutation Ser90Gly appears to be a common feature (Figure 1). Ser90 is located at the beginning of α4 helix and its mutation to Gly residue may influence its flexibility/conformation, which in turn triggers structural changes in α2 helix. This can be achieved through the contribution of the intersubunit lock-and-key motif, which is a conserved structural motif in phi class GSTs [14,38]. In this motif, the protruded Phe52 (the “key”) interacts through several non-polar interactions with amino acids (e.g., Trp101, Val104, Thr108, Val149, Tyr150, the “lock”) located at the middle of the α4 and α5 helices of the neighbor subunit. The alterations of structure/flexibility of the α4 helix induced by the Ser90Gly mutation are transmitted through Phe52 to α2 helix (Figure 10a).

Normal mode analysis allowed the calculation of deformation energy that provides an estimation of protein local flexibility, while the atomic fluctuation shows the amplitude for the absolute atomic motion [62]. Figure 10b–d show the protein local flexibility and fluctuation for the parent enzyme and sh155, confirming the differences in dynamics.

#### 2.5.2. Structural Elements That Contribute to Thermostability

**Figure 11:**
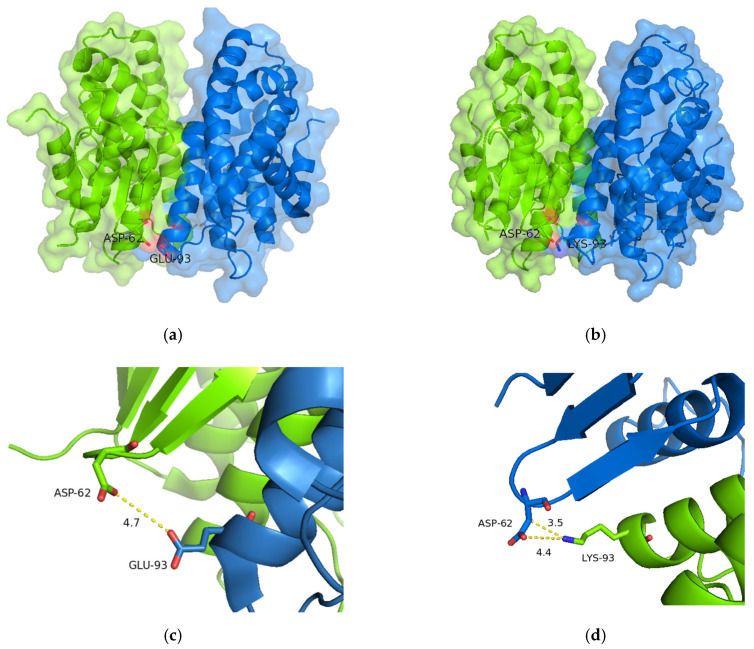

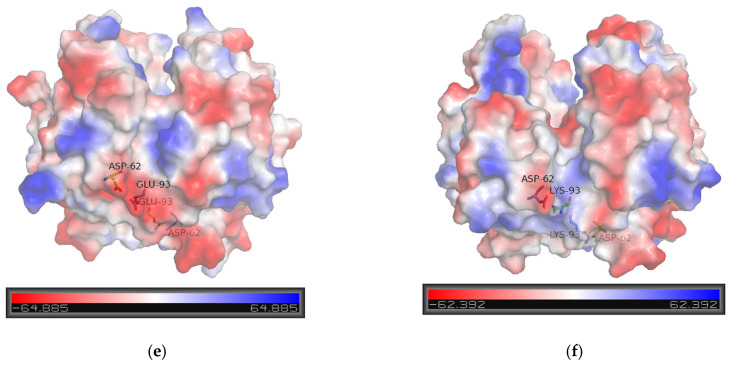
Amino acid residue at position 93 in *Am*GSTF (Glu93; (**a**,**c**,**e**)) and sh101 (Lys; (**b**,**d**,**f**)), which is involved in the formation of a new salt bridge with Asp62 of the opposite subunit. (**e**,**f**) Electrostatic surface of the dimers is depicted with 50% transparency. The figures were created with PyMol [61].

Amino acid sequence alignments and structural analysis revealed crucial amino acids that contribute to structural stability. Considering the results listed in Table 2, it is conceivable that the parent enzymes and the variants may be clustered into two groups. The first group contains the variants sh101, sh155, sh168 and the parent enzymes *Lr*GSTF, *Td*GSTF and *Hv*GSTF that display high thermostability (T_m_ > 65 °C), while the other group contains sh49, sh152 and the parent enzyme *Am*GSTF. Amino acid sequence alignment (Figure 1b) shows that in all thermostable enzymes, Glu93 has been substituted for Lys. Inspection of the crystal structures of sh101 and sh155 (Figure 11) shows that Lys93 can form a new salt bridge with Asp62 of the opposite subunit. This new electrostatic interaction can presumably provide a significant stabilization effect to the dimeric structure. Notably, the less thermostable enzymes (i.e., sh49, sh152 and the parent enzyme *Am*GSTF) lack the Glu93Lys mutation, allowing the non-favorable interaction between Glu93 and Asp62 (Figure 11).

#### 2.5.3. Structural Elements That Contribute to Inhibition Potency

**Figure 12:**
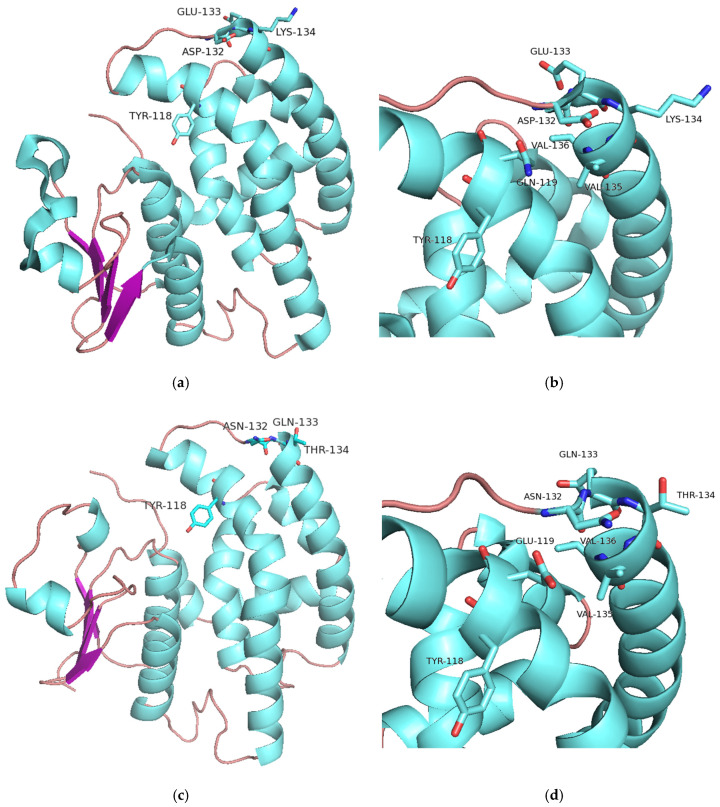
(**a**) Ribbon representation of the *Am*GSTF monomer, showing the motif Asp-Glu-Lys in relation to Tyr118. (**b**) Amino acid residues in *Am*GSTF that contribute towards inhibition sensitivity by butachlor. (**c**) Ribbon representation of the monomer of sh155 variant, showing the Asn-Gln-Thr motif. (**d**) Amino acid residues in sh155 variant that contribute towards inhibition sensitivity by butachlor. The figures were created by PyMol [61].

Recently, a specific 3D pharmacophore targeting the MHR-GSTFs was designed and used to identify structural elements important for their potent and selective inhibition [31]. In this previous work, structural analysis of GSTFs revealed a decisive role of Tyr118 in ligand binding and pharmacophore design. Its positioning is dependent on an outer patch of adjacent residues that span from position 132 to 134. In *Lr*GSTF and *Am*GSTF, the sequence is composed by Asp-Glu-Lys, whereas in *Hv*GSTF and *Td*GSTF by Asn-Gln-Thr (Figure 1b). Considering the IC_50_ values (Table 2), it is obvious that the shuffled enzyme variants which possess the Asp-Glu-Lys sequence (e.g., sh49, sh168) are more sensitive to inhibition compared to the enzymes that have the Asn-Gln-Thr sequence (sh155, sh152, sh101, sh12, sh147). The loss of Asp-Glu-Lys motif seems to destabilize the optimal orientation of Tyr118 and thus significantly alters the sensitivity of *Hv*GSTF and *Td*GSTF to the given compound (Figure 12). Furthermore, the amino acid at position 119 appears to contribute to the inhibition potency. This amino acid interacts with the conserved Val135-Val136 hydrophobic patch, which is located on the α5 helix. The position and integrity of the α5 helix contributes to the orientation of the α4, which provides the important residues Tyr118, Phe122 that determine the structure of the H-site and regulates k_cat_. Notably, in the sequence of more sensitive enzymes the amino acid at position 119 is Gln, whereas it has been replaced by Glu in the less sensitive enzymes. In addition, the inhibition potency is also linked with the identity of the amino acid at position 122 (Phe vs. Ile) and 125 (Met vs. Leu). All these data suggest that a concerted crosstalk between different structural elements on the polypeptide chain determine the inhibition sensitivity of GSTFs.

## 3. Materials and Methods 

### 3.1. Materials

The pETite C-His expression vector was included in the Expresso™ T7 Cloning and Expression System (Lucigen, Middleton, WI, USA). KAPA Taq and KAPA High Fidelity DNA polymerases were obtained from KAPA Biostystems (KAPA Biostystems Pty, Cape Town, South Africa). RNase-Free DNase (Promega, Madison, WI, USA) and the restriction enzyme, Dpn1, were used (Invitrogen, Carlsbad, CA, USA). Cloning was achieved using the In-Fusion^®^ HD Cloning Kit (Takara Bio USA Inc, Mountain View, CA, USA). The mini prep plasmid isolation and the gel extraction kits were purchased from Macherey-Nagel (Macherey-Nagel GmbH & Co., Düren, Germany). All enzyme substrates, antibiotics and analytical grade salts were obtained from Sigma-Aldrich (Sigma-Aldrich Co., St. Louis, MO, USA).

### 3.2. Methods

#### 3.2.1. Preparation of DNA Shuffling and Construction of GSTF Library

Amplification of parental *gstf* genes was performed using KAPA HiFi DNA polymerase. Primers were designed in order to generate PCR products containing flanking homologous overlaps to the pEXP5-CT/TOPO vector’s sequence, allowing homologous recombination between the identical sites (Table S1). The DNA template for *T. durum* and *H. vulgare* GSTFs (*Td*GSTF, *Hv*GSTF) was harbored in pEXP5-CT/TOPO vector, whereas *A. myosuroides* and *L. rigidum* GSTFs (*Am*GSTF, *Lr*GSTF) were synthetic genes purchased from Eurofins Genomics, dissolved in appropriate buffer. All reactions were conducted in a final volume of 50 μL consisting of 0.3 mM of each dNTP, 15 pmol of forward and reverse primers, 1 ng DNA template, 5X KAPA HiFi Buffer and 0.5 Units KAPA HiFi DNA polymerase. Initial denaturation was performed at 95 °C for 3 min. A total of 30 cycles of denaturation at 98 °C for 20 s, annealing at different temperatures according to each gene for 15 s and extension at 72 °C for 1 min, followed by 10 min at 72 °C. The annealing temperature for the genes of *Am*GSTF and *Td*GSTF was 67 °C, for *Hv*GSTF 62 °C and for *Lr*GSTF 64 °C. The PCR products were analyzed on a 1% (*w*/*v*) agarose gel. The products corresponding to *Td*GSTF and *Hv*GSTF genes were excised and purified using Macherey-Nagel’s Nucleospin^®^ Gel and PCR Clean-up kit (Macherey-Nagel GmbH & Co., Düren, Germany). They were also treated with the restriction enzyme Dpn1 to ensure the absence of any parental plasmid. The reaction was incubated in 10X Buffer (33 mM Tris-acetate, pH 7.9, 10 mM magnesium acetate, 66 mM potassium acetate, 0.1 mg/mL BSA) and 1.5 Unit Dpn1 for 3 h at 37 °C, followed by 20 min at 80 °C.

The applied method of DNA shuffling for in vitro directed evolution was based on various previous publications [39,40,41,63,64,65]. DNA fragmentation was achieved using DNase and equal proportions of the amplified genes in a final volume of 40 μL. The reaction containing 19.2 μL of an equal part DNA mixture, 4 µL 10X DNase buffer (400 mM Tris-HCl, pH 8, 100 mM MgSO_4_, 10 mM CaCl₂) and 16.8 µL of sterile ddH₂O or TE Buffer, was initially equilibrated for 5 min at 15 °C. After the addition of 0.7 Units of DNase, the mixture was equilibrated for a total of 15 min at 15 °C. Digestion was stopped in small aliquots at different time points, using 20 mM EDTA, pH 8, and incubation at 65 °C for 10 min. The fragmentation process was evaluated by 2% (*w*/*v*) agarose gel electrophoresis. Random DNA fragments of 50–100 bp were obtained at the time span of 8 to 15 min of the reaction.

The recovered DNA fragments were subjected to PCR without the addition of external primers (reassembling reaction). The reaction contained 3 μL of 5X KAPA HiFi Buffer, 0.3 mM of each dNTP, a total of 9 μL of DNA fragments and 0.3 Units HiFi DNA Polymerase at a final volume of 15 μL. The program used in the thermocycler consisted of 3 min initial denaturation at 95 °C, a total of 35 cycles of denaturation at 98 °C for 20 s, annealing at 55 °C for 15 s, extension at 72 °C for 1 min and finally another 10 min extension at 72 °C. Therefore, consecutive PCRs were conducted using primer pairs corresponding to each gene in order to amplify the reassembled products. These primers were designed to allow homologous recombination and subcloning in the pETite C-His vector (Table S1). Each reaction had a final volume of 25 µL and contained 12.5 µL CloneAmp™ HiFi PCR Premix, 10 pmol forward primer, 10 pmol reverse primer and 1 µL 1:10 diluted PCR product of the reassembling reaction. Initial denaturation was conducted at 98 °C for 4 min. A total of 35 cycles of denaturation at 98 °C for 10 s, annealing at temperatures corresponding to each gene for 15 s and extension at 72 °C for 5 s, was followed by 10 min at 72 °C. The annealing temperature for *Lr*GSTF was 65 °C, 66 °C for *Hv*GSTF and 67 °C for *Am*GSTF and *Td*GSTF. The PCR products were evaluated by 1% (*w*/*v*) agarose gel electrophoresis and the anticipated bands between 600 and 700 bp were extracted. The purified products were cloned into pETite C-His vector, following the procedure of the In-Fusion^®^ HD Cloning Kit, and were transformed into *E. coli* Stellar. These were spread on LB agar plates containing kanamycin (30 μg/mL). Hundreds of transformants were grown in LB medium at 37 °C containing kanamycin (30 μg/mL) and their enzymatic activities were assessed using the CDNB/GSH substrate system.

#### 3.2.2. GSTF Expression and Purification Methods

Expression of the recombinant GSTF mutants was based on previously published procedures with modifications for optimization [30,66]. *E. coli* strains harboring recombinant pETite C-His vectors were cultured for 24 h at 37 °C in appropriate medium (0.2% (*w*/*v*) lactose, 0.5% (*w*/*v*) glycerol, 1% (*w*/*v*) peptone (tryptone), 0.5% (*w*/*v*) yeast extract, 0.05% (*w*/*v*) glycose, 0.07% (*w*/*v*) sodium sulphate, 0.25% (*w*/*v*) ammonium chloride and 0.01% (*w*/*v*) of calcium chloride, potassium chloride and magnesium chloride) containing 30 μg/mL kanamycin along with 34 μg/mL chloramphenicol. The strains utilized were BL21 (DE3) pLysS, though Rosetta™ 2 (DE3) pLysS were used specifically for the mutants sh12, sh101, sh147, sh152. Furthermore, sh12 and sh101 mutants exhibited slightly increased expression when cultured in LB medium in the presence of suitable antibiotics at 37 °C overnight. Isopropyl 1-thio-β galactopyranoside (IPTG, 1 mM) was added when their absorbance at 600 nm was 0.5–0.6. Four hours after induction, cells were harvested by centrifugation at 8000 rpm for 10 min, resuspended in lysis buffer (50 mM NaH₂PO_4_, 300 mM NaCl, 10 mM imidazole, pH 8), sonicated and centrifuged at 13,000 rpm for 5 min. Enzyme purification was achieved via Ni-IDA-Sepharose affinity chromatography as previously described [30,59]. Protein purity was evaluated by SDS-PAGE.

#### 3.2.3. Enzyme Activity and Kinetic Analysis Assays

Enzyme activity assays for the CDNB conjugation reactions were performed according to previously published methods [40,46,67]. Initial velocities were determined at least in triplicate in 0.1 M sodium phosphate buffer, pH 6.5, equilibrated at 37 °C with final concentrations of 2.5 mM GSH and 1 mM CDNB. Turnover numbers were calculated based on the notion of one active site per subunit. One unit of enzyme activity is defined as the amount of enzyme that catalyzes the conversion of 1 micromole of substrate per min in specified conditions (1 U = 1 μmol/min). Peroxidase activity assays were also performed using cumene hydroperoxide (CuOOH) and tert-butyl hydroperoxide (tert-BuOOH) as substrates [68,69]. Specific activity was expressed in micromoles per minute per milligram of protein. Protein concentration was determined by the Bradford assay using bovine serum albumin for the formation of the standard curve [70].

Kinetic analysis was performed as described in earlier publications [39,46] in 0.1 M sodium phosphate buffer, pH 6.5, equilibrated at 37 °C. Michaelis–Menten and allosteric sigmoidal equations were fitted as needed to the steady-state data by nonlinear regression analysis using GraphPad Prism version 7 (GraphPad Prism Software Inc., San Diego, CA, USA).

#### 3.2.4. Protein Thermal Stability: T_50_ and T_m_ Determination

Thermal inactivation of purified GSTFs was measured in 20 mM potassium phosphate buffer, pH 7, after incubating for five minutes at a temperature range of 10–80 °C. Residual activity was determined, considering as 100% the enzyme’s activity at 4 °C. T_50_ (defined as the temperature where 50% of the initial enzyme activity is lost after stated heat treatment conditions) was determined by fitting the Boltzmann sigmoidal equation to the residual activity and temperature.

Thermal stability of purified GSTFs was further investigated by the thermal shift assay according to published methods [71]. Thermal denaturation of proteins was determined using a Real-time PCR StepOne™ instrument (Applied Biosystems, Waltham, MA, USA) and the SYPRO Orange protein dye. The thermal stability assay was carried out in 20 mM potassium phosphate buffer, pH 7.0, and the fluorescence monitoring took place at a temperature range between 15 °C and 99 °C with a ramping rate of 1%. Melting temperatures (T_m_) were calculated by nonlinear fitting of the Boltzmann equation to the melt region normalized fluorescence data. T_m_ is defined as the denaturation midpoint of a protein, hence the temperature where 50% of the protein is unfolded.

#### 3.2.5. Crystallization and Structure Determination

Sh101 and sh155 were concentrated to 10 mg/mL and 12 mg/mL, respectively, in buffer HEPES 10 mM, NaCl 100 mM, NaN_3_ 0.002%, pH 7.0. Crystals were produced with the hanging-drop vapor diffusion technique. Sh101 crystals were grown in condition 26 of MIDAS crystallization screen (Molecular Dimensions, Sheffield, UK; 0.2 M sodium chloride; 0.1 M MES, pH 6; 30% *v*/*v* Jeffamine ED-2003). Sh155 crystals were grown under two conditions: (i) HEPES-NaOH 0.1 M, PEG 4000 20% *w*/*v*, 2-propanol 10% *v*/*v*, pH 7.5; (ii) Ammonium sulphate 0.2 M, PEG 4000 15–17.5% *w*/*v*, pH 7.8. X-ray diffraction data for sh101 and sh155 were collected under cryogenic conditions with 20% glycerol as cryoprotectant on the P13 beamline at EMBL-Hamburg (c/o DESY) and BioMAX (MAX IV), respectively. Structure determination was carried out by molecular replacement in Phaser [72] using the structure of *Am*GSTF (pdb id 6riv) as template (~92% sequence identity). Sh155 crystals grown in condition (ii) were used for structure determination. Refinement was carried out with Phenix v. 1.20.1-4487 [73] and rebuilding and visualization of the structures with Coot v. 0.9 [74]. X-ray data collection and refinement statistics are shown in Supplementary Materials Table S2.

#### 3.2.6. Structural Analysis

The structures were analyzed using PyMol [61] and UCSF Chimera [55]. Normal mode dynamics of the parent enzyme and the sh155 variant were studied using the tools implemented in DynaMut [62]. Initial sequence alignment was conducted by Clustal Omega [44] and analyzed by ESPript 3.0 [43]. GSTF protein models were created by Swiss-Model [75].

## 4. Conclusions

In this study, a library of mutant GSTFs was created by in vitro directed evolution via DNA shuffling. Kinetic and structural analysis of wild-type and selected enzyme variants resulted in the identification of new GSTFs with improved catalytic properties and thermal stability. Furthermore, the crystal structure of mutant sh101 and sh155, which demonstrated the most improved catalytic parameters and thermal stability, highlighted significant structural elements related to substrate and inhibitor binding and catalysis. Important structural elements include: (a) the amino acid residues Phe122, Met125 that affect k_cat_, (b) the unfavorable interaction between Glu93 and Asp62 side chains that influences the thermostability, and (c) the amino acid patch 132–134, which, in connection with Gln119, contributes towards the inhibition sensitivity for butachlor. These new GSTFs hold significant potential for utilization in a variety of biotechnology applications as sustainable biocatalysts.

## Figures and Tables

**Table 1:** Steady-state kinetic parameters of wild-type GSTFs and enzyme variants for the CDNB/GSH substrate system. Kinetic analysis was performed at 37 °C and pH 6.5. The measurements were performed in triplicate and the data represent the mean ± SD (N = 3). The parameters k_cat_/K_m_ and k_cat_/S_0.5_ were calculated by the established mean values.

	K_m_ (mM)	S_0.5_ (mM)	n_H_	k_cat_ (min^−1^)	k_cat_/K_m_ (min^−1^ mM^−1^)	k_cat_/S_0.5_ (min^−1^ mM^−1^)
	*GSH*	*CDNB*			*GSH*	*CDNB*
*Am*GSTF	1.78 ± 0.04	0.65 ± 0.03	1.5 ± 0.05	35.9 ± 1.7	20.17	55.23
*Lr*GSTF	1.36 ± 0.04	0.76 ± 0.04	1.41 ± 0.05	28.35 ± 1.02	20.85	37.30
*Hv*GSTF	0.8 ± 0.017	0.52 ± 0.01	1.46 ± 0.03	110 ± 3.05	137.5	211.54
*Td*GSTF	0.9 ± 0.03	0.51 ± 0.02	1.43 ± 0.06	141.3 ± 5.08	157	277.06
sh12	1.4 ± 0.04	0.35 ± 0.01	1.16 ± 0.03	151.2 ± 1.9	108	432.00
sh49	1.5 ± 0.05	0.61 ± 0.02	1.41 ± 0.05	34.8 ± 0.5	23.2	57.05
sh101	1.37 ± 0.05	0.41 ± 0.02	1.1 ± 0.02	185.9 ± 1.8	135.69	453.4
sh147	1.4 ± 0.03	0.46 ± 0.03	1.05 ± 0.03	149.6 ± 1.8	106.86	325.20
sh155	1.43 ± 0.04	0.77 ± 0.06	1.02 ± 0.03	197.7 ± 3.18	138.25	256.75
sh152	1.3 ± 0.05	0.38 ± 0.02	1.13 ± 0.04	140.4 ± 2.1	108.00	369.47
sh168	1.7 ± 0.05	0.85 ± 0.04	1.28 ± 0.04	34.05 ± 0.9	20.03	40.10

**Table 2:** Summary of IC_50_ values for butachlor on the parent GSTFs and enzyme variants. The data represent the mean ± SD (N = 3).

**Parent GSTFs**							
	***Td*** **GSTF**	***Hv*** **GSTF**	***Lr*** **GSTF**	***Am*** **GSTF**			
Butachlor (μM)	9.9 ± 0.2	9.2 ± 0.6	7.1 ± 0.5	7.3 ± 0.4			
**Shuffled enzyme variants**							
	**sh12**	**sh49**	**sh101**	**sh147**	**sh152**	**sh155**	**sh168**
Butachlor (μM)	6.8 ± 0.1	2.6 ± 0.04	5.9 ± 0.4	9.4 ± 0.7	4.4 ± 0.07	3.4 ± 0.1	3.1 ± 0.08

**Table 3:** Summary of T_m_ and T_50_ values as determined by thermal shift assay and thermal inactivation studies for the parent GSTFs and shuffled enzyme variants. The data represent the mean ± SD (N = 3). The T_m_ − T_50_ was calculated by the established mean values.

	T_50_ (°C)	T_m_ (°C)	T_m_ − T_50_ (°C)
***Am*GSTF**	61.4 ± 0.2	62.8 ± 0.04	1.4
***Td*GSTF**	69 ± 0.9	71.1 ± 0.05	2.1
***Hv*GSTF**	66.8 ± 0.2	69.5 ± 0.05	2.7
***Lr*GSTF**	67.5 ± 0.2	70.2 ± 0.04	2.7
**sh12**	63.7 ± 0.2	65.4 ± 0.04	1.7
**sh49**	58 ± 0.1	60.1 ± 0.04	2.1
**sh101**	69.3 ± 0.3	71.1 ± 0.06	1.8
**sh147**	63.3 ± 0.3	64.5 ± 0.05	1.2
**sh152**	58.8 ± 0.1	60.8 ± 0.03	2
**sh155**	66.6 ± 0.3	68.0 ± 0.05	1.4
**sh168**	65.8 ± 0.2	66.8 ± 0.06	1.0

## Data Availability

The data presented in this study are available in the article and in the Supplementary Materials.

## Supplementary Materials

The following supporting information can be downloaded at: https://www.mdpi.com/article/10.3390/ijms23137469/s1.
