# Supplementary material for: Directed Evolution of Phi Class Glutathione Transferases Involved in Multiple-Herbicide Resistance of Grass Weeds and Crops
Source: Int J Mol Sci. 2022 Jul 5;23(13):7469. doi: 10.3390/ijms23137469 (PMC9267659; doi:10.3390/ijms23137469)
Supplement: Supplementary file 1 [file ijms-23-07469-s001.zip › ijms-1776411-supplementary.pdf]

SUPPLEMENTARY MATERIALS

Table S1: Primer sequences that were used in this study.

Primer Sets		Nucleotide Sequence
**pEXP5-CT/TOPO homologous overlaps**		
***Am*GSTF**	**Forward**	5' GAAGGAGATACCCTTATGGCACCTGTAAAAGTCTTTGGT 3'
	**Reverse**	5' GTGATGATGACCCTT-CGCTTTTGGCGGAACCATTGT 3'
***Hv*GSTF**	**Forward**	5' GAAGGAGATACCCTTATGGCTCCGGTGAAGGTG 3'
	**Reverse**	5' GTGATGATGACCCTTCGGCTTCTTGGGAACCATCTG 3'
***Lr*GSTF**	**Forward**	5' GAAGGAGATACCCTTATGGCGCCAGTGAAAGTGTTTG 3'
	**Reverse**	5' GTGATGATGACCCTTGGCTTTCGGTGGAACCATCGTTG 3'
***Td*GSTF**	**Forward**	5'GAAGGAGATACCCTTATGGCGCCGGTGAAGGTGTTCG 3'
	**Reverse**	5'GTGATGATGACCCTTCGGCTTCTTGGGAACCATTTGC 3'
**pETite C-His homologous overlaps**		
***Am*GSTF**	**Forward**	5' GAAGGAGATATACATATGGCACCTGTAAAAGTCTTTGGTC 3'
	**Reverse**	5' GTGATGGTGGTGATGATGCGCTTTTGGCGGAACCATTG 3'
***Hv*GSTF**	**Forward**	5' GAAGGAGATATACATATGGCTCCGGTGAAGGTGTTC 3'
	**Reverse**	5' GTGATGGTGGTGATGATGCGGCTTCTTGGGAACCATCTG 3'
***Lr*GSTF**	**Forward**	5' GAAGGAGATATACATATGGCGCCAGTGAAAGTGTTTGG 3'
	**Reverse**	5' GTGATGGTGGTGATGATGGGCTTTCGGTGGAACCATCG 3'
***Td*GSTF**	**Forward**	5' GAAGGAGATATACATATGGCGCCGGTGAAGGTGTTC 3'
	**Reverse**	5' GTGATGGTGGTGATGATGCGGCTTCTTGGGAACCATTTG 3'
**pETite C-His**		
**Forward**		5' CATCATCACCACCATCACTA 3'
**Reverse**		5' CATATGTATATCTCCTTCTTATAGT 3'

Table S2. X-ray data collection and refinement statistics.

**Data collection**	**Sh101**	**Sh155**
Beamline	P13 (EMBL, Hamburg)	BioMAX (MAX IV, Lund)
Wavelength (Å)	0.9763	0.9918
Resolution (Å)	45.97-1.87 (1.93-1.87)	83.10-2.05 (2.11-2.05)
Space group	*C*2	*I*2
Unit cell (Å)<br>*a, b, c* (Å)<br>β (°)	 92.01, 46.23, 110.25<br>112.29	 46.48, 99.00, 153.21<br>93.73
No. Molecules/asymmetric unit	2	3
No. of observations	241684 (23609)	212204 (16765)
No. of unique reflections	36297 (3552)	43427 (3415)
I/sigma(I)	10.9 (0.7)	10.4 (1.0)
Completeness (%)	99.8 (99.9)	99.0 (99.0)
Multiplicity	6.7 (6.6)	4.9 (4.9)
Mosaicity (°)	0.22	0.10
R_{meas}	0.087 (2.934)	0.118 (2.255)
$CC_{1/2}$	0.9999 (0.28)	0.998 (0.456)
Wilson B factor (Å^2)	47.9	35.2
**Refinement**		
No. of reflections used	36206	43336
R_{cryst}/R_{free}	0.196/0.231	0.216/0.252
RMSD in bonds (Å)	0.008	0.008
RMSD in angles (°)	0.97	0.92
No. of water molecules	101	147
Average B-factor (Å^2)	54.1	52.5
Ramachandran favored/outliers (%)	95.4/1.53	93.6/0.9
Clashscore	4.53	7.05
PDB id	7ZA5	7ZA4

Table S3. GSTFs protein models created by the Swiss-Model.

	Sequence Identity	*Template*	*GMQE*	*QMEANDisCo Global*
sh12	92.24	6riv.1.A	0.91	0.9 ± 0.05
sh49	97.26	6riv.1.A	0.92	0.9 ± 0.05
sh147	91.78	6riv.1.A	0.91	0.89 ± 0.05
sh152	94.52	6riv.1.A	0.91	0,9 ± 0.05
sh168	94.52	6riv.1.A	0.92	0.9 ± 0.05

SUPPLEMENTARY FIGURES

	1	2	3	4	5	6	7	8	9	10	11
LrGSTF	100.00	90.95	91.32	91.78	88.24	89.14	88.13	87.67	89.04	89.04	89.50
AmGSTF	90.95	100.00	97.26	94.52	87.33	88.24	92.24	91.78	94.52	94.98	92.24
sh49	91.32	97.26	100.00	97.26	89.95	90.87	91.32	91.78	92.69	92.24	94.98
sh168	91.78	94.52	97.26	100.00	91.78	89.95	93.15	93.61	91.32	91.32	94.06
TdGSTF	88.24	87.33	89.95	91.78	100.00	96.83	94.98	95.43	91.32	91.78	94.52
HvGSTF	89.14	88.24	90.87	89.95	96.83	100.00	91.78	92.24	91.78	93.15	95.89
sh12	88.13	92.24	91.32	93.15	94.98	91.78	100.00	99.54	95.43	96.80	95.89
sh147	87.67	91.78	91.78	93.61	95.43	92.24	99.54	100.00	94.98	96.35	96.35
sh152	89.04	94.52	92.69	91.32	91.32	91.78	95.43	94.98	100.00	96.80	94.98
sh101	89.04	94.98	92.24	91.32	91.78	93.15	96.80	96.35	96.80	100.00	97.26
sh155	89.50	92.24	94.98	94.06	94.52	95.89	95.89	96.35	94.98	97.26	100.00

Figure S1: Sequence identity (%) between the parent and the enzyme variants that were studied in the present work.

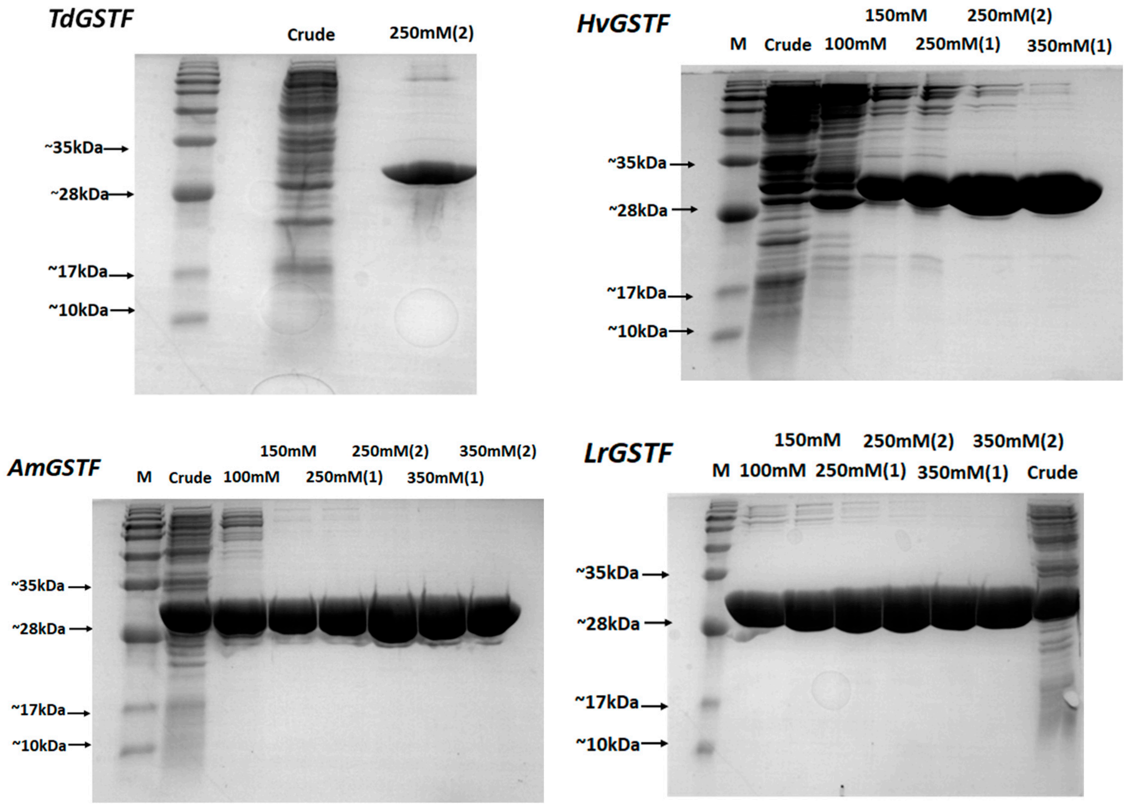

Figure S2. SDS-PAGE analysis of the purification of the parent GSTF enzymes by Ni-IDA-Sepharose affinity chromatography. “M” denotes the molecular marker, “Crude” the enzyme extract before purification. The concentration of imidazole (mM) that was used in each eluted fraction is showing above each gel.

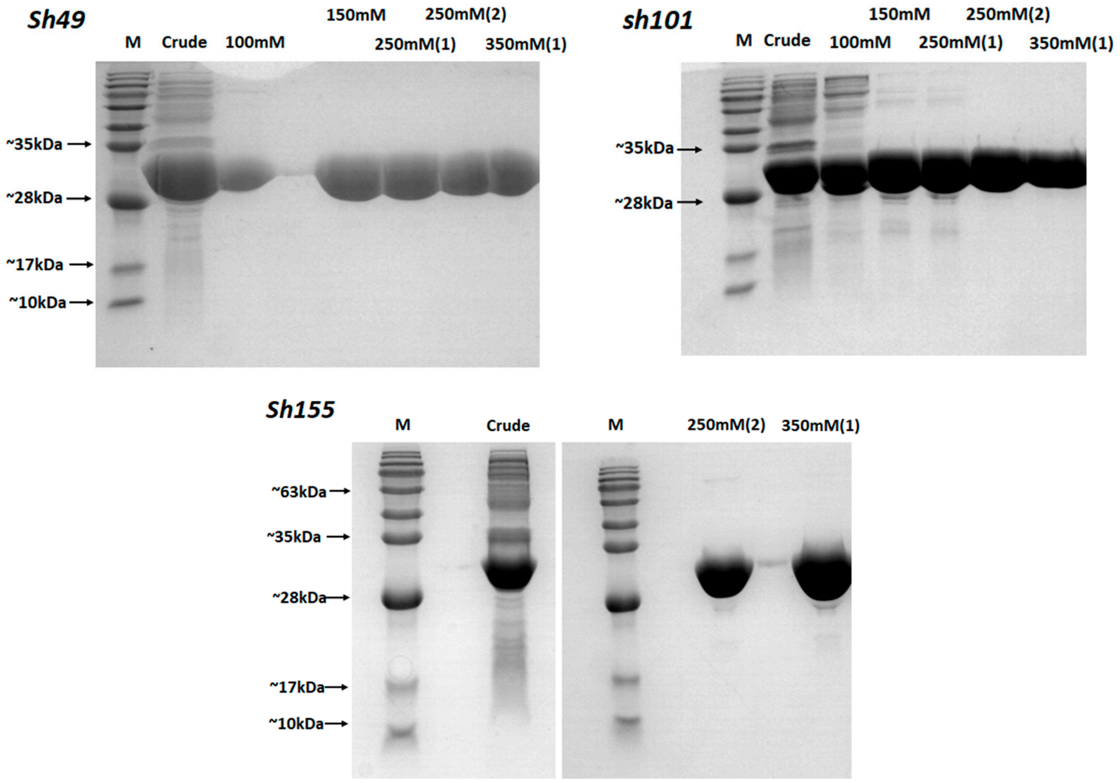

Figure S3. SDS-PAGE analysis of the purification of selected enzyme variants (sh49, sh101, sh155) by Ni-IDA-Sepharose affinity chromatography. "M" denotes the molecular marker, "Crude" the enzyme extract before purification. The concentration of imidazole (mM) that was used in each eluted fraction is showing above each gel.

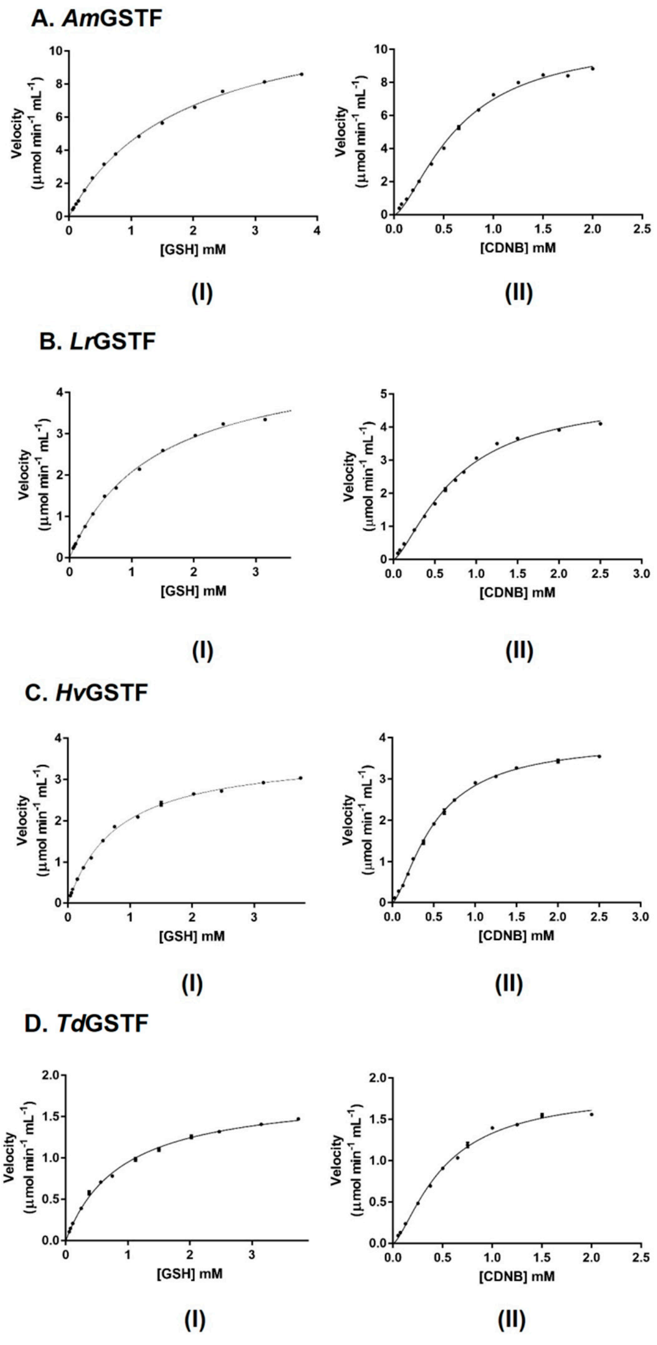

Figure S4. Steady-state kinetics analysis of the parent GSTFs. Steady-state kinetic analysis using GSH as a variable substrate and CDNB at a fixed concentration [A-D: (I)]. Steady-state kinetic analysis using the CDNB as a variable substrate and GSH at a fixed concentration [A-D: (II)]. The measurements were performed in triplicate and the data represent the mean ± SD (N=3).

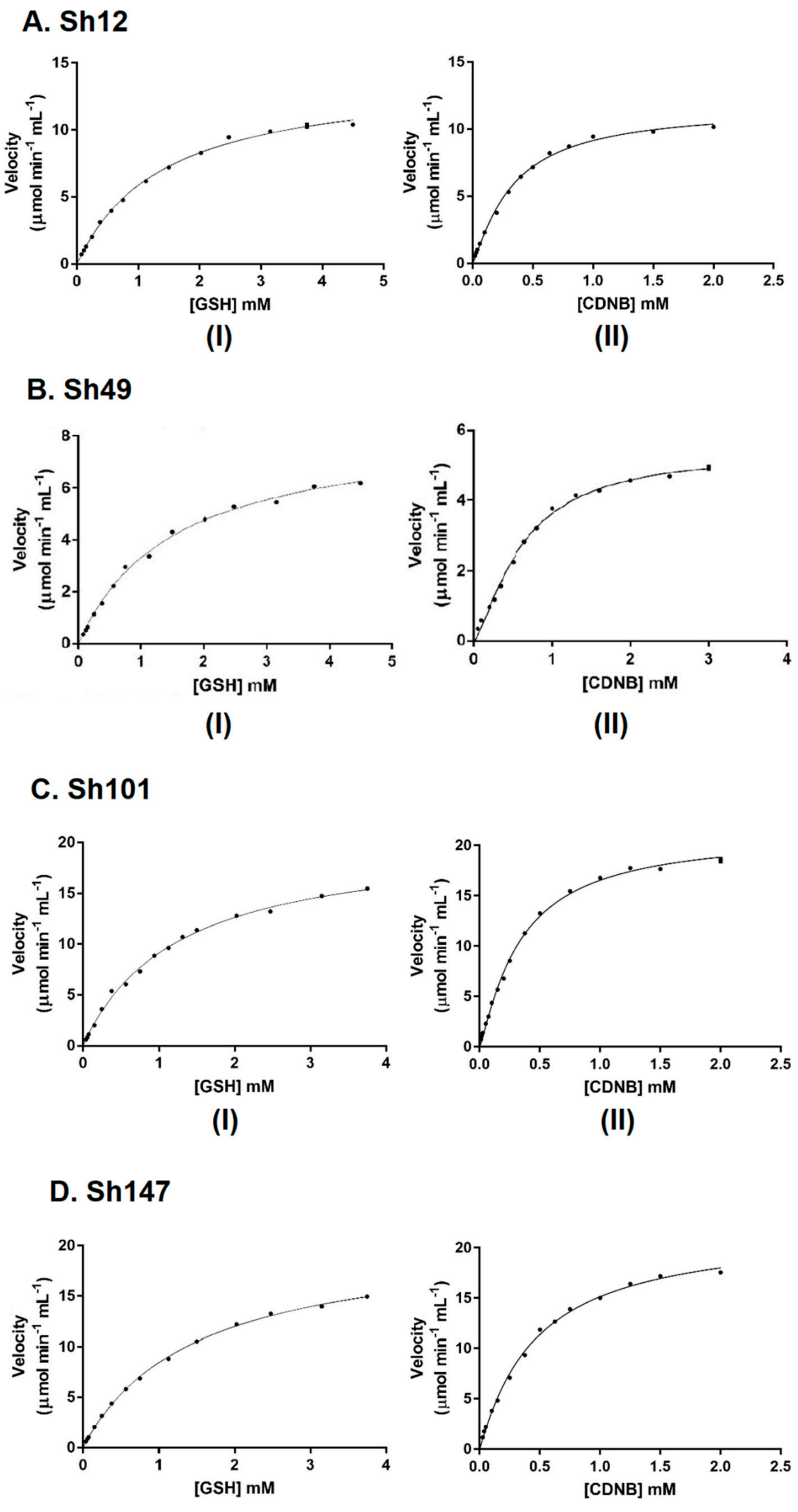
A. Sh12
Velocity (μmol min-1 mL-1)
[GSH] mM
[CDNB] mM
(I)
(II)
B. Sh49
Velocity (μmol min-1 mL-1)
[GSH] mM
[CDNB] mM
(I)
(II)
C. Sh101
Velocity (μmol min-1 mL-1)
[GSH] mM
[CDNB] mM
(I)
(II)
D. Sh147
Velocity (μmol min-1 mL-1)
[GSH] mM
[CDNB] mM

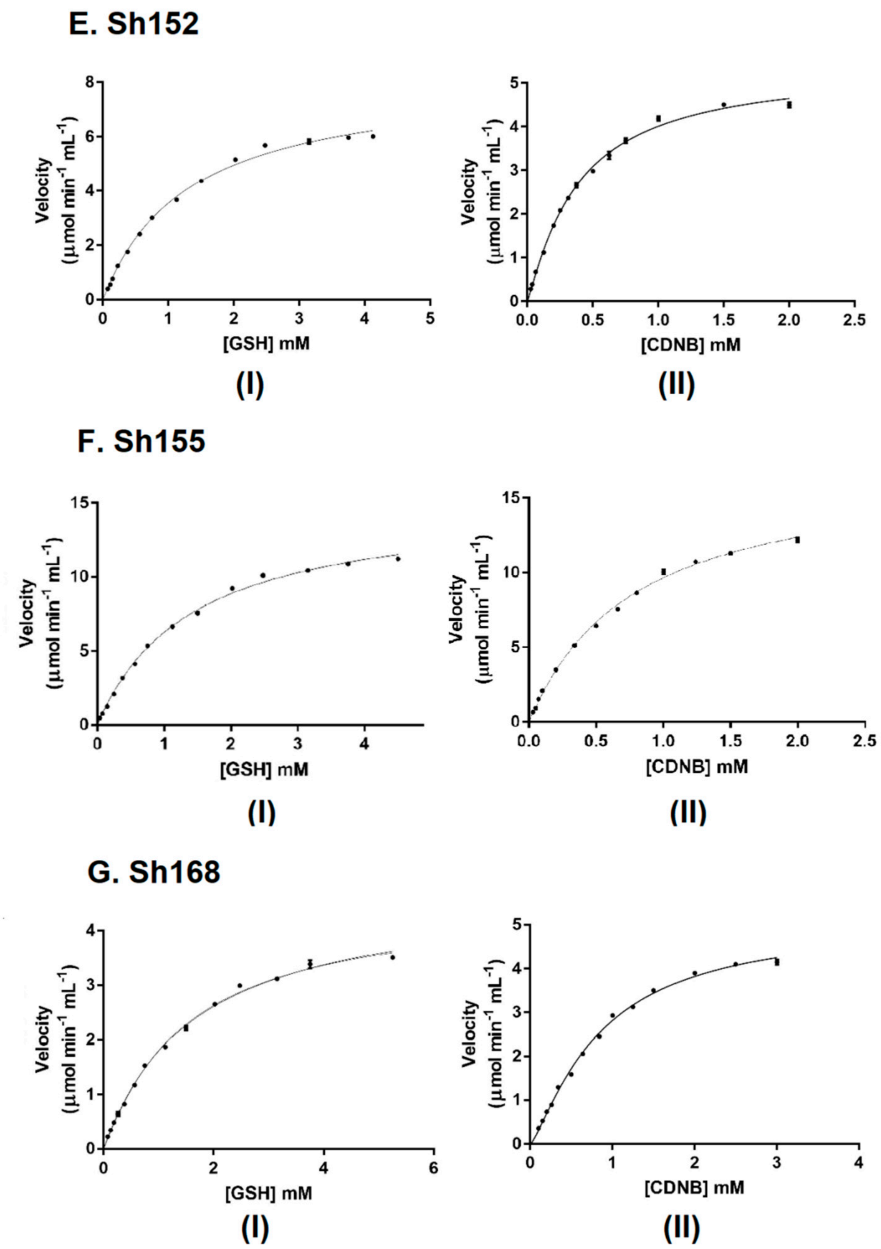

Figure S5. Steady-state kinetics analysis of the DNA Shuffled enzyme variants. Steady-state kinetic analysis using GSH as a variable substrate and CDNB at a fixed concentration [A-G: (I)]. Steady-state kinetic analysis using the CDNB as a variable substrate and GSH at a fixed concentration [A-G: (II)]. The measurements were carried in triplicate and the data represent the mean ± SD (N=3).
